# Intraoperative Cardiac Arrest Triggered by Carbon Dioxide Insufflation During Laparoscopic Surgery: A Case Report

**DOI:** 10.7759/cureus.106594

**Published:** 2026-04-07

**Authors:** Nirajan Khati, Rakesh K Shah, Sammi Joshi, Subodh Chapagain, Pragnesh H Gadhvi

**Affiliations:** 1 Internal Medicine, Jersey City Medical Center, Jersey City, USA; 2 Internal Medicine, Detroit Medical Center/Sinai Grace Hospital, Detroit, USA; 3 Internal Medicine, Nepal Medical College, Kathmandu , NPL; 4 Internal Medicine, Kathmandu Medical College, Kathmandu, NPL; 5 Cardiology, Jersey City Medical Center, Jersey City, USA

**Keywords:** asystole, bradyarrhythmia, co2 insufflation, intraoperative cardiac arrest., laparoscopic surgeries, pneumoperitoneum, reduced venous return, vagal tone

## Abstract

Minimally invasive laparoscopic surgeries, favored for their reduced postoperative pain and quicker recovery times, may pose intraoperative hemodynamic risks due to the creation of pneumoperitoneum. This case report presents an episode of sudden cardiac arrest following carbon dioxide (CO2) insufflation during an elective robotic-assisted multispecialty endometrial excision in a female patient in her 40s. Despite preoperative assessment indicating low perioperative risk, the patient experienced severe bradycardia, progressing rapidly to asystole after CO2 insufflation. The case report emphasizes the importance of early detection and timely intervention for cardiac complications during laparoscopic surgeries.

## Introduction

Laparoscopic surgeries are popular due to less postoperative pain, faster recovery, and shorter hospital stays. The overall mortality rate for laparoscopic surgery is low, ranging from 0.3% to 1.8% [1,2]. Carbon dioxide (CO2) is cost-effective, non-combustible, highly soluble, and quickly exhaled through respiration, making it safer and more efficient for creating pneumoperitoneum during laparoscopic surgery [3]. However, pneumoperitoneum during laparoscopic surgery decreases cardiac output by up to 30% due to reduced venous return while increasing systemic vascular resistance [4]. Abdominal insufflation during laparoscopic surgery stretches the peritoneum, increasing vagal tone and potentially leading to bradyarrhythmia or even asystole. In healthy individuals, bradyarrhythmia occurs in 14% to 27% of cases [5]. There are a few cases reported of cardiac arrest during the creation of pneumoperitoneum during laparoscopic surgeries. This case study highlights an instance of intraoperative cardiac arrest triggered by CO2 insufflation in a female in her 40s undergoing gynecological laparoscopic surgery.

## Case presentation

A female patient in her 40s with a past medical history significant for hypothyroidism, irritable bowel syndrome (IBS), and endometriosis was scheduled for an elective robotic-assisted multispecialty endometrial excision. Per-operative evaluation deemed her a low risk for perioperative complications. Pre-op vitals include blood pressure (BP) 103/68, heart rate (HR) 66, respiratory rate (RR) 18, afebrile, and peripheral oxygen saturation (SpO2) 100% on room air (RA). Initial placement of the laparoscopic ports and CO2 insufflation was uneventful. Intra-abdominal pressure (IAP) was maintained at 12 mmHg with an insufflation rate of 2 L/min. Shortly after the insufflation began, the patient experienced a sudden and profound drop in heart rate to 30 bpm, followed by complete asystole within a few minutes. Immediate actions included cessation of CO2 insufflation and repositioning of the patient. The anesthesia team promptly initiated cardiopulmonary resuscitation (CPR). They administered two doses of intravenous epinephrine as part of the advanced cardiovascular life support (ACLS) protocol. After approximately 6 minutes of cardiopulmonary resuscitation, the patient achieved a return of spontaneous circulation (ROSC) and was successfully stabilized and intubated. Following ROSC, the patient was transferred to the intensive care unit for further management and close monitoring. She remained intubated and sedated to facilitate hemodynamic stability and was gradually weaned off sedation and extubated on postoperative day one.

**Figure 1:**
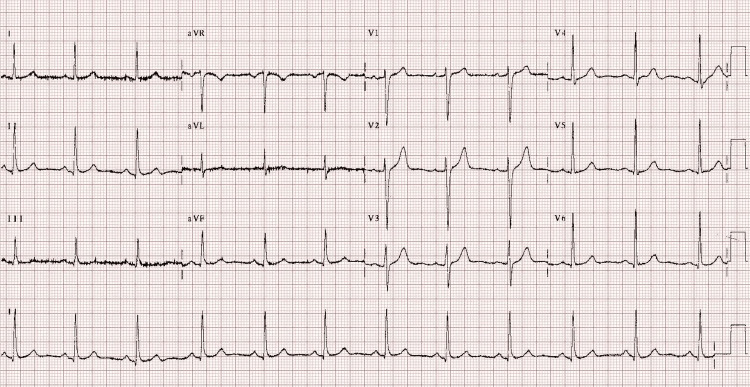
EKG showing normal sinus rhythm with no acute ischemic changes

**Figure 2:**
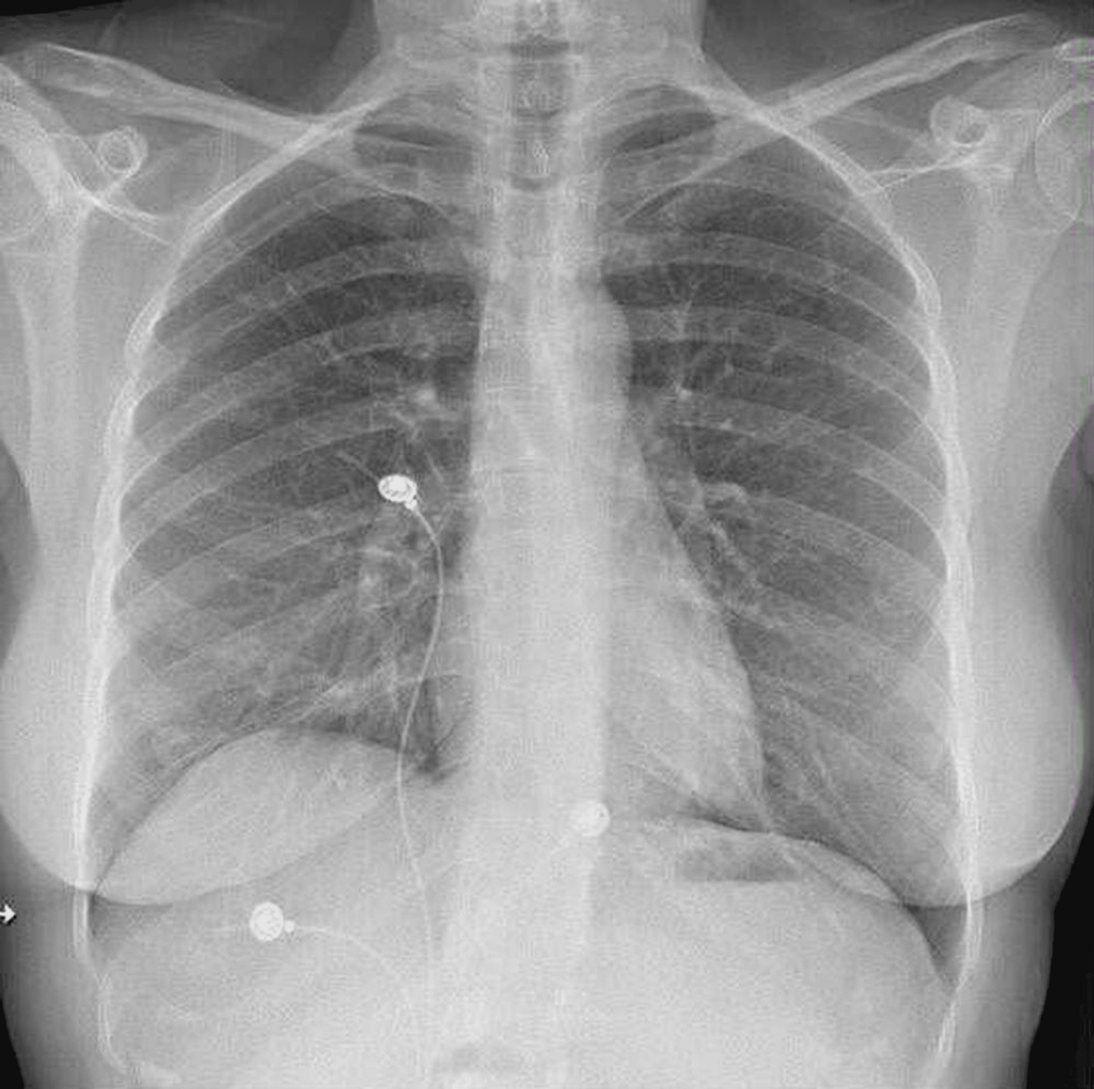
Chest X-ray showing no acute cardiopulmonary findings

**Figure 3:**
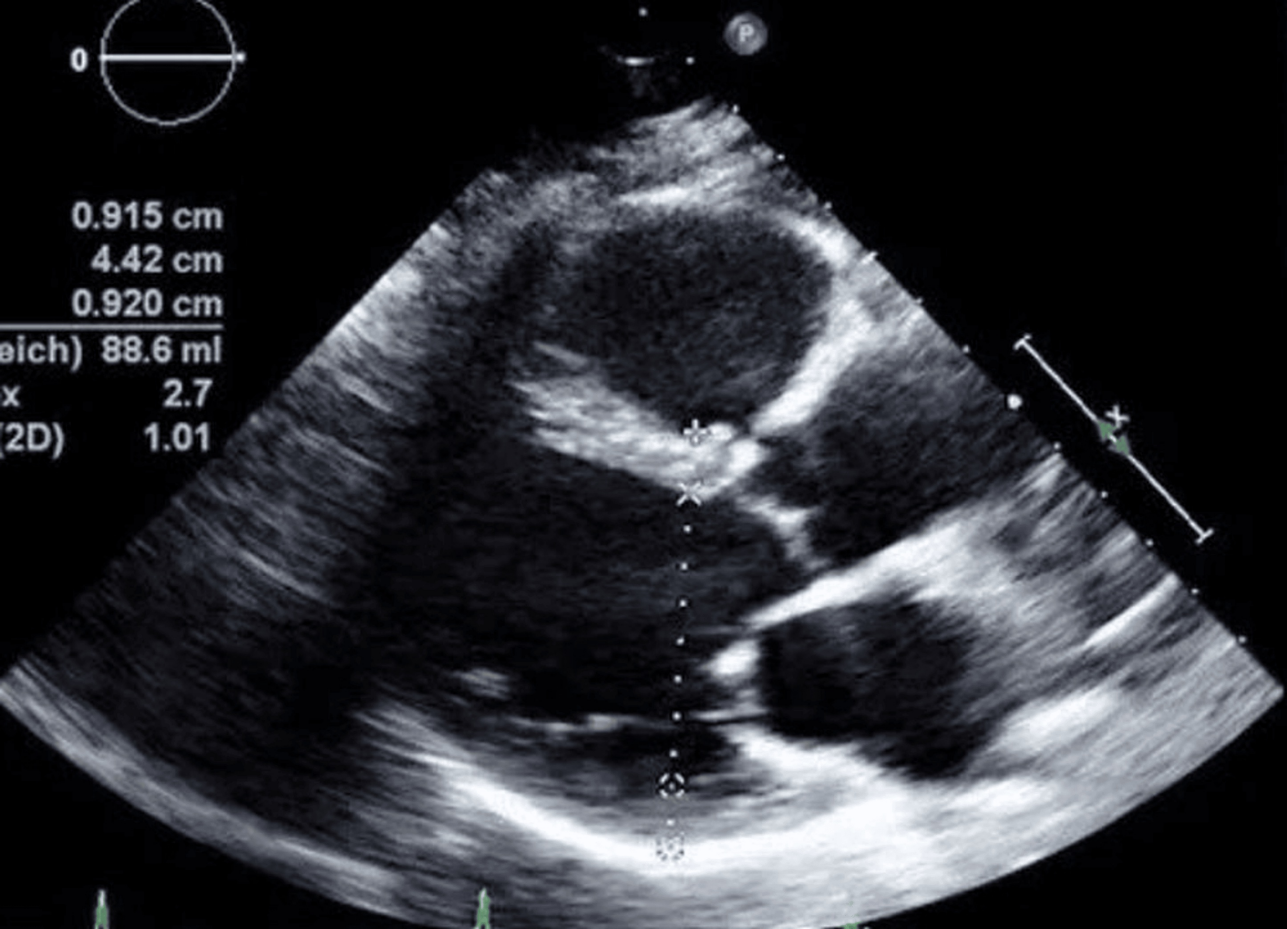
Transthoracic echocardiogram (TTE) showed normal left ventricular ejection fraction (LVEF) and no evidence of regional wall motion abnormalities (RWMA)

Post-cardiac arrest workup showed potassium 4.2, B-type natriuretic peptide (BNP) 96, and serial troponin of 2784>2236 ng/L, as shown in Table 1; this value steadily decreased over time, suggesting transient myocardial stress or a non-ischemic cause of the troponin elevation likely from post CPR. EKG was unremarkable for any acute ischemic changes, as in Figure 1. A chest X-ray did not show any acute cardiopulmonary findings, as shown in Figure 2. Point-of-care ultrasound (POCUS) performed immediately after the event, followed by a formal transthoracic echocardiogram (TTE), demonstrated normal left ventricular function without evidence of regional wall motion abnormalities, as shown in Figure 3.

**Table 1:** Lab results of the patient BNP: B-type natriuretic peptide, TC: total cholesterol, TG: triglycerides, LDL: low-density lipoprotein, HbA1c: glycated hemoglobin, TSH: thyroid-stimulating hormone

Parameters	Results	Reference range
Sodium	138	135-145 mmol/L
Potassium	4.2	3.5-5.1 mmol/L
Magnesium	2.1	1.6-2.6 mg/dl
Phosphorus	3	2.4-5.1 mg/dl
Troponin I High Sensitivity	2784>2236	3-34 ng/L
BNP	96	2-100 pg/ml
White count	6	4.5-11x10*3 /uL
TC/TG/LDL	210/190/108	TC:25 - 200 mg/dl, TG:10 -150 mg/dl, LDL:5-100mg/dl
HbA1c	5.2	3.8 - 5.7 %
TSH	2.2	0.55 - 4.78 mIU/L

Cardiology was consulted. Further evaluation by the cardiology team included reviewing her pre-existing conditions and potential predisposing factors for such a severe vagal response. The patient had no prior history of vagal episodes, syncope, or presyncope. No definitive intrinsic cardiac pathology was identified, supporting the theory that an exaggerated autonomic response to intra-abdominal pressure could have triggered the event. The cardiologist recommended outpatient follow-up with Holter monitoring and possibly an electrophysiological study to identify any latent conduction disorders that might predispose her to bradyarrhythmia or vagal overactivity.

## Discussion

CO2 is the preferred gas for pneumoperitoneum because of its low combustibility and high solubility in blood, which lowers the risk of gas embolism [4]. Still, it has effects on the body's physiology and may cause harm. The two key factors of CO2 insufflation that affect the cardio-pulmonary system are increased intra-abdominal pressure and hypercarbia. Increased intra-abdominal pressure from CO2 insufflation reduces venous return, compressing the vena cava and decreasing cardiac output [5]. Additionally, CO2 insufflation can trigger a vagal response, leading to bradycardia and, in severe cases, even asystole [6]. According to the study published by Youg J et al., 14 cases of cardiac arrest occurred during laparoscopic gynecological surgery, mostly in healthy patients. Twelve out of those 14 were linked to pneumoperitoneum, with bradycardia preceding arrest in 75% of cases [7]. Makkieh N in 2017 described a case of a healthy 25-year-old female who sustained intra-operative cardiac arrest following bradycardia a few minutes after CO2 insufflation [8]. Another mechanism by which patients can undergo cardiac arrest during laparoscopic surgery is by CO2 embolism, as reported in a study by Kim NS, who highlighted cardiac arrest due to CO2 embolism during laparoscopy gynecology surgery [9].

Insufflation with a high flow rate while establishing artificial pneumoperitoneum instantaneously increases IAP, and unexpected cardiovascular changes, such as hypotension and bradyarrhythmia, occur. In contrast, a slow rate of insufflation prevents such complications. In patients with compromised cardiorespiratory status, such a vagal response with abrupt and rapid insufflations of CO2 can prove to be detrimental. Therefore, not only maintaining IAP below 12-15 mmHg but also keeping a slow insufflation rate (2-4lit/min) when establishing pneumoperitoneum is important and essential [10].

Management of complications includes immediate cessation of CO2 insufflation and repositioning to relieve pressure, followed by the advanced cardiovascular life support (ACLS) protocol, demonstrating the effectiveness of prompt action in critical situations [11]. Cho EJ et al. described two case reports on cardiac arrest after gas insufflation for laparoscopic surgery with successful recovery in both cases following cardiac resuscitation [12]. Postoperatively, the patient should be monitored in the intensive care unit, with serial troponin measurements that may indicate transient myocardial stress rather than ischemic injury. Post-cardiac arrest EKG can help assess any acute ischemic changes or QT interval abnormalities. An echocardiogram can help assess the ventricular function and regional wall motion abnormalities [13].

Recent advancements in surgical and anesthetic techniques have led to guidelines focused on optimizing hemodynamic management during laparoscopic surgeries. Recommendations by the Anesthesia Patient Safety Foundation (APSF) highlight early detection of hemodynamic instability, thorough preoperative risk assessment, and the use of advanced monitoring technologies [14]. Novel strategies, such as local anesthetic infiltration, have been explored to reduce reflex bradycardia. At the same time, patient positioning and the careful management of insufflation pressure have shown potential to prevent complications during laparoscopies [15]. A multidisciplinary approach involving anesthesia providers, surgeons, and perioperative teams is essential to address patient-specific risks and intervene promptly.

## Conclusions

This case illustrates the critical need for vigilant cardiovascular monitoring during laparoscopic surgeries, particularly in patients with known risk factors. Slow insufflation and close monitoring are crucial to prevent cardiovascular collapse during laparoscopic surgery. This approach helps minimize the risk of complications like bradycardia and cardiac arrest. Prompt management of intraoperative bradycardia is essential, as it can be an early warning sign of impending cardiac arrest. The prompt recognition and management of intraoperative complications, as demonstrated in this case, are essential for improving patient outcomes and preventing catastrophic events.
